# B-mode ultrasound common carotid artery intima-media thickness and external diameter: cross-sectional and longitudinal associations with carotid atherosclerosis in a large population sample

**DOI:** 10.1186/1476-7120-6-10

**Published:** 2008-03-05

**Authors:** Marsha L Eigenbrodt, Zoran Bursac, Richard E Tracy, Jawahar L Mehta, Kathryn M Rose, David J Couper

**Affiliations:** 1 Department of Epidemiology, Fay W. Boozman College of Public Health, University of Arkansas for Medical Sciences, Little Rock, AR, USA; 2 Department of Biostatistics, Fay W. Boozman College of Public Health, University of Arkansas for Medical Sciences, Little Rock, AR, USA; 3 Department of Pathology, Louisiana State University Health Science Center, New Orleans, LA, USA; 4 Departments of Internal Medicine, Physiology, and Biophysics, University of Arkansas for Medical Sciences, Little Rock, AR, USA; 5 Department of Epidemiology, School of Public Health, University of North Carolina at Chapel Hill, NC, USA; 6 Department of Biostatistics, University of North Carolina at Chapel Hill, NC, USA

## Abstract

**Background:**

Arterial diameter and intima-media thickness (IMT) enlargement may each be related to the atherosclerotic process. Their separate or combined enlargement may indicate different arterial phenotypes with different atherosclerosis risk.

**Methods:**

We investigated cross-sectional (baseline 1987–89: n = 7956) and prospective (median follow-up = 5.9 years: n = 4845) associations between baseline right common carotid artery (RCCA) external diameter and IMT with existing and incident carotid atherosclerotic lesions detected by B-mode ultrasound in any right or left carotid segments. Logistic regression models (unadjusted, adjusted for IMT, or adjusted for IMT and risk factors) were used to relate baseline diameter to existing carotid lesions while comparably adjusted parametric survival models assessed baseline diameter associations with carotid atherosclerosis progression (incident carotid lesions). Four baseline arterial phenotypes were categorized as having 1) neither IMT nor diameter enlarged (reference), 2) isolated IMT thickening, 3) isolated diameter enlargement, and 4) enlargement of both IMT and diameter. The association between these phenotypes and progression to definitive carotid atherosclerotic lesions was assessed over the follow-up period.

**Results:**

Each standard deviation increment of baseline RCCA diameter was associated with increasing carotid lesion prevalence (unadjusted odds ratio [OR] = 1.54, 95% confidence interval [CI] = 1.47–1.62) and with progression of carotid atherosclerosis (unadjusted hazards ratio (HR) = 1.37, 95% CI = 1.28–1.46); and the associations remained significant even after adjustment for IMT and risk factors (prevalence OR = 1.11, 95% CI = 1.04–1.18; progression HR = 1.11, 95% CI = 1.03–1.19). Controlling for gender, age and race, persons with both RCCA IMT and diameter in the upper 50^th ^percentiles had the greatest risk of progressing to clearly defined carotid atherosclerotic lesions (all HR = 1.71, 95% CI = 1.47–2.0; men HR = 1.88, 95% CI = 1.48–2.39; women HR = 1.59, 95% CI = 1.31–1.95) while RCCA IMT or diameter alone in the upper 50^th ^percentile produced significantly lower estimated risks.

**Conclusion:**

RCCA IMT and external diameter provide partially overlapping information relating to carotid atherosclerotic lesions. More importantly, the RCCA phenotype of coexistent wall thickening with external diameter enlargement indicates higher atherosclerotic risk than isolated wall thickening or diameter enlargement.

## Background

Risk factors contribute to atherosclerosis through gradual arterial changes that may produce ischemia by either progressive luminal narrowing or more commonly, by sudden plaque rupture or intimal erosions with formation of an in situ occlusive thrombus [1]. A widely accepted, convenient marker of atherosclerosis is carotid artery intima-media thickness (IMT) [2] which is significantly associated with prevalent [3,4] and incident [5] carotid plaques. While a number of factors can contribute to error in ultrasound artery measurements [6,7], variation in the progression of atherosclerosis at different arterial sites, and not error in ultrasound measurements, is thought to contribute to some discrepancies in the prediction of coronary events [8]. However, arterial parameters other than IMT may provide insights into how risk factors are related to different stages of atherosclerosis [9-11], promote an understanding of arterial segment differences [12], or provide understanding of how classification based on carotid ultrasound and coronary angiography may differ [13]. Since arterial wall area incorporates both diameter and wall thickness, area estimation may provide some advantages to IMT alone [14,15]. If different cardiovascular risk factors are associated with disparate changes in IMT and diameter, or the parameter changes are manifested at different stages of disease progression, then considering both measures jointly may identify the atherosclerotic phenotypes more effectively [13,16-18]. The relationship of plaques, IMT, and artery diameter is complex and a number of arterial phenotype classifications have been proposed [18-24]. Risk factors are associated with arterial wall thickness [25], IMT progression [26-28], artery diameter [29-31], and calcified carotid plaques [32]. Correlations between carotid IMT and diameter (0.31 to 0.59) [29,31,33] vary across populations and may depend upon whether the internal or external diameter [31] is evaluated. Part of the correlation may reflect an adaptive process used to maintain arterial wall stress [33-35], but in the presence of vulnerable atherosclerotic plaques, arterial diameter may reflect direct damage of the internal elastic lamina and arterial media [36,37]. So, risk factors may contribute to IMT and diameter directly and indirectly.

The current study suggests that combined wall thickening and diameter enlargement indicates a higher risk arterial phenotype than either isolated abnormality. This may be relevant to the pathobiology of atherosclerosis.

## Methods

### Study sample

The ARICLAD (Atherosclerosis Risk in Communities Limited Access Data) is a subset of the ARIC Study database (N = 15792) [38] limited to participants whose informed consent agrees to data sharing (n = 15732, 99.6%). The sampling strategy for the ARIC Cohort Study has been reported previously [39], and the ARIC Study procedures are available [38]. The current study was approved by the University of Arkansas for Medical Sciences Institutional Review Board. In brief, this study uses data collected primarily at clinical exams that recurred on average at 3 year intervals from the 1987–89 baseline exams through the fourth exam cycle that ended in 1998. The ARIC participants (15792 black and non-black men and women, ages 45 to 64 at baseline) were recruited from four centers in the U.S. as previously described [39]. The ARICLAD was divided randomly into developmental and test datasets (10000 and 5732 persons, respectively) to be used in several studies. For this study, participants missing the following baseline information were excluded: status of plaques/shadowing at any carotid site (n = 3915), RCCA diameter, IMT, measures needed for calculation of arterial wall area (circular or elliptical) (n = 2576), or model 3 covariates (n = 1285). After exclusions, 7956 participants remained, of which 5015 in the developmental subset were used to develop cross-sectional models. Excluding participants with baseline plaques/shadowing (n = 2955) and those who had no follow-up data on plaques/shadowing (n = 156) left 4845 participants of which 3060 were used to develop the models of atherosclerosis progression with incident carotid lesions as the outcome. Potential covariates included race, gender, and baseline age, height, current smoking status, cigarette years of smoking (based on years of smoking and numbers of cigarettes smoked per day), current drinking status, usual ethanol consumption (grams per week calculated from self-reported usual drinks per week), body mass index (BMI = weight in kilograms/height in meters^2^), diabetes status, blood glucose, cholesterol medication use, systolic and diastolic brachial blood pressure (mm Hg, means of second and third sitting measurements), anti-hypertensive medication use, fibrinogen, HDL- and LDL-cholesterol, peripheral white blood count, and physical activity (sport index) [40].

### B-mode ultrasound

B-mode ultrasound scans were performed at baseline and at exam 2 on most participants and on overlapping subsets of participants at exams 3 and 4 [38]. Detailed ultrasound methods can be found on the ARIC Limited Access Data Navigation System under Ultrasound Manuals. The CCA IMT and external diameter (interadventitial distance) measures, as defined by ARIC in the "optimal' view were the primary independent variables investigated. Because of more complete information on the right than the left CCA, the right-sided measures were primary and the left-sided measures were secondary. Plaques were not intentionally excluded from IMT and diameter measurements and likely contribute to variability of measurements. Presence of carotid atherosclerotic lesions (plaques or shadowing) was determined from scans of all right and left carotid artery segments (CCA, bifurcation, and internal carotid artery) [41]. The presence of plaques was defined during ultrasound reading based on wall thickness and arterial wall roughness, loss of alignment, or protrusion into the lumen [42]. Calcification or mineralization, another indicator of atherosclerosis, was based on acoustic shadowing (shadowing) [41]. For the current study, a carotid atherosclerotic lesion was defined as missing if any of the six carotid sites had missing data for plaque/shadowing status and another carotid site was not positive. Because relatively complete information from all six carotid sites at baseline (including the CCA, bulb and the internal carotid segments) was required to construct the carotid lesion variable, a substantial number of participants (N = 3915) had missing baseline information.

### Right CCA wall areas

RCCA wall area was calculated as the total artery area minus the lumen area assuming a circular lumen and an outer artery structure that was either circular or elliptical. The formula *A *= *πr*^2 ^- *π*(*r *- *IMT*)^2 ^where *A *is the arterial wall area, *r *is the artery radius, and IMT is wall thickness was used to estimate wall area assuming circular configurations [14,15]. Wall area calculations based on an elliptical outer artery structure were performed as previously described [43].

### Statistical Methods

All analyses were performed using SASv9 (SAS Institute Inc., Cary, NC). Two multivariable adjustment methods, logistic regression and parametric time-to-event models allowing for interval censoring (SAS LIFEREG procedure assuming the Weibull distribution of event time), were the main analytical tools. Associations between one standard deviation (SD) increments of the baseline vascular measures with plaques/shadowing were assessed for the full sample and gender subsets. The unadjusted model (model 1) included RCCA IMT, diameter, wall area, or both IMT and diameter (IMT+diameter). Model 2 added race, age, height, and gender (in the overall model). Two risk factor adjusted models were used: model 4 included all 20 covariates while a more parsimonious model (model 3) included only covariates identified by stepwise logistic regression analyses as significant (p < 0.05) in at least one of the vascular measure models. To investigate the association of arterial diameter enlargement with atherosclerosis progression, we excluded persons with carotid lesions at baseline and assessed whether baseline RCCA diameter predicted the development of readily identifiable new plaques or shadowing in any carotid site during follow-up. Covariates were similarly selected in the prospective models as in the cross-sectional model. See tables for model 3 covariates. The c-statistic for logistic regression models was used to assess individual discrimination for each model.

**Figure 1:**
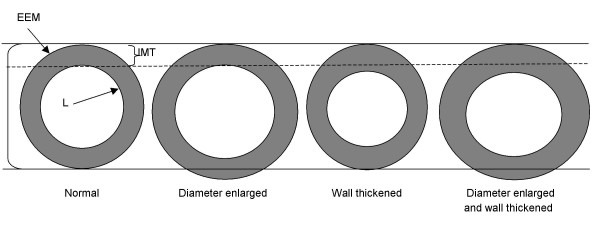
Representation of four arterial phenotypes based on enlargement of arterial wall thickness and external diameter. EEM = interface of media and adventitia. IMT = indicates normal intima-media thickness. L = interface of intima with lumen. Large bracket indicates normal external diameter.

We also investigated the association of four arterial phenotypes at baseline with carotid atherosclerosis progression. The baseline arterial phenotypes consisted of isolated or concurrent enlargement of RCCA diameter and IMT or neither (See Figure 1). The 50^th ^percentile value of each vascular parameter for men and women was used to classify persons as having an enlarged RCCA IMT (men: ≥0.66 mm; women: ≥0.61 mm) and/or diameter (men: ≥8.06 mm; women: ≥7.30 mm). To check the robustness of this approach, separate categorizations were based on the observed to expected ratio values for IMT and diameter. Expected IMT and diameter values were calculated for each participant based on gender-specific betas for age, race, and height determined from linear regression analyses performed on a subset of participants who were free of atherosclerotic-related conditions (carotid atherosclerotic lesions, stroke, coronary heart disease, diabetes mellitus, hypertension [definition includes use of medication], ever smokers, use of cholesterol lowering medication, BMI ≥ 30, or LDL-cholesterol >160 mg/dl) at baseline. The observed/expected ratios for the RCCA parameter were ranked and persons in the upper 50^th ^percentile were classified as having the RCCA measure enlarged.

## Results

Out of 20 tested risk factors and characteristics only one, diabetes status, was statistically significantly (p < 0.05) different between the developmental and test datasets (8.8% in the developmental and 10.3% in the test data set). This difference is modest, and while significant, is consistent with what would be expected by chance.

Overall, 2955 (37.1%) participants had atherosclerotic lesions in at least one carotid segment at baseline. Compared to participants without plaques/shadowing (Table 1), participants with lesions were older and were more likely to be current and former smokers, former drinkers, diabetics, and use anti-hypertensive and cholesterol-lowering medications. Lifetime abstainers from smoking and drinking were more common among those without baseline carotid lesions. Among current users, the reported quantity of cigarettes smoked over their lifetime and usual ethanol consumption was higher among persons with than those without carotid lesions. Persons with lesions were also found to have less favorable levels of systolic blood pressure, LDL-cholesterol, HDL-cholesterol, fibrinogen, white blood count, and blood glucose compared to persons without lesions; while diastolic blood pressure, and sport index were similar in both groups. BMI and the proportion of black participants were higher among persons without than among those with carotid lesions. After adjusting for gender, persons with carotid lesions were shorter than those without lesions. Height- and gender-adjusted RCCA wall thickness, diameter and calculated wall areas were larger among persons with than among those without carotid lesions.

**Table 1:** Characteristics of all participants and of subsets with and without carotid lesions at baseline, Atherosclerosis Risk in Communities Limited Access Data, 1987–89.

		Full Study	Carotid Lesions:		
		Sample	Present	Absent	
		N = 7956	N = 2955	N = 5001	p-value
Age, years (mean (SD))		54.0 (5.7)	55.7 (5.5)	53.0 (5.6)	<0.0001
Male Gender (%)		43.4	52	38.3	<0.0001
Black race (%)		22.6	21.1	23.4	0.02
Smoker (%)	Current	25.6	31.7	21.9	<0.0001
	Former	31.4	34.2	29.8	
	Never	43.0	34.2	48.3	
Cigarette years* (mean (SD))		660 (418)	735 (449)	596 (379)	<0.0001
Drinker (%)	Current	58.5	58.6	58.4	0.0002
	Former	17.3	19.3	16.1	
	Never	24.2	22.0	25.4	
Ethanol, grams/week^† ^(mean (SD))		72 (111)	79 (125)	60 (102)	<0.0001
Diabetes (%)		8.8	10.3	7.9	0.0002
Hypertension medication (%)		24.6	36.5	26.8	<0.0001
Cholesterol medication (%)		2.8	3.4	2.4	0.011
Body Mass Index (mean (SD))		26.7 (4.6)	26.5 (4.4)	26.8 (4.7)	0.0032
Systolic BP, mm Hg (mean (SD))		120 (18)	123 (19)	118 (17)	<0.0001
Diastolic BP, mm Hg (mean (SD))		72.7 (10.9)	73.0 (11.0)	72.6 (10.9)	0.146
LDL-C, mmol/L (mean (SD))		3.53 (1.00)	3.69 (0.98)	3.44 (1.01)	<0.0001
HDL-C, mmol/L (mean (SD))		1.38 (0.45)	1.32 (0.43)	1.41 (0.46)	<0.0001
Fibrinogen, mg/dL (mean (SD))		298 (63)	305 (64)	294 (62)	<0.0001
White blood count, 1000s/μL (mean (SD))		6.0 (1.9)	6.3 (2.0)	5.9 (1.8)	<0.0001
Blood glucose, mg/dL (mean (SD))		105 (33)	107 (36)	104 (31)	<0.0001
Sport index (mean (SD))		2.47 (0.80)	2.48 (0.79)	2.47 (0.81)	0.34
Standing height, cm (mean (SE)) ^‡^		168.5 (0.1)	169 (0.11)	169.6 (0.09)	<0.0001
RCCA measures (mean (SE)) ^§^					
Intima-medial thickness (mm)		0.65 (0.002)	0.71 (0.003)	0.63 (0.002)	<0.0001
Diameter (mm)		7.72 (0.01)	7.94 (0.01)	7.65 (0.01)	<0.0001
Circular wall area (mm^2^)		14.6 (0.05)	16.2 (0.08)	13.9 (0.06)	<0.0001
Elliptical wall area (mm^2^)		20.9 (0.06)	22.8 (0.09)	20.1 (0.07)	<0.0001

BP = blood pressure.

IMT = intima-media thickness.

RCCA = right common carotid artery.

SD = standard deviation.

SE = standard error.

N = Maximum number possible. Lower numbers were available for some variables because of missing information.

* Among current smokers.

^†^Among current drinkers.

^‡^Height is adjusted for gender in the carotid lesion subsets.

^§ ^RCCA measures are adjusted for height and gender in the carotid lesion subsets.

In unadjusted cross-sectional models, SD units of IMT (OR = 1.75), diameter (OR = 1.54), and calculated circular RCCA wall area (OR = 1.83) were significantly associated with increased prevalence of carotid atherosclerosis (Table 2). Adjustment for demographic factors and height (model 2) or statistically significant risk factors (model 3) reduced the strength of associations as did inclusion of both RCCA IMT and diameter in the same model; however IMT, diameter and wall area all remained statistically significant (P < 0.05) at all levels of adjustment in the full sample and in the gender subsets. Inclusion of all 20 risk factors did not further reduce the odds ratios and all vascular measures remained statistically significant (data not shown). Since IMT was used by ARIC in defining plaques [42], the robust association of IMT with carotid lesions was not unexpected. The stronger RCCA wall area association with prevalent carotid lesions among women compared to men in unadjusted analyses (model 1) were no longer significantly different after controlling for atherosclerotic risk factors (model 3).

**Table 2:** Cross-sectional associations* between 1-standard deviation (SD) increments of B- mode ultrasound right common carotid artery (RCCA) measures and prevalent carotid atherosclerotic lesions for the full sample and by gender, Atherosclerosis Risk in Communities Limited Access Data, 1987–1989.

		Odds Ratio (95% Confidence Interval)		
		Full Sample	Men	Women
RCCA Measure	Adjustment Level^†^	N = 7956	N = 3453	N = 4503
IMT	Model 1	1.75 (1.66–1.84)	1.67 (1.55–1.80)	1.72 (1.59–1.86)
	Model 2	1.54 (1.46–1.53)	1.53 (1.41–1.65)	1.56 (1.44–1.69)
	Model 3	1.46 (1.38–1.55)	1.46 (1.34–1.58)	1.47 (1.36–1.60)
Diameter	Model 1	1.54 (1.47–1.62)	1.41 (1.31–1.51)	1.55 (1.43–1.67)
	Model 2	1.36 (1.29–1.44)	1.32 (1.23–1.42)	1.41 (1.30–1.53)
	Model 3	1.24 (1.17–1.32)	1.25 (1.15–1.35)	1.25 (1.15–1.37)
Wall area	Model 1	1.83 (1.73–1.92)	1.68 (1.56–1.81)	1.85 (1.71–2.01)
	Model 2	1.61 (1.52–1.70)	1.54 (1.43–1.66)	1.69 (1.55–1.84)
	Model 3	1.49 (1.41–1.59)	1.47 (1.36–1.59)	1.54 (1.41–1.69)

	Model 1			
IMT		1.56 (1.47–1.65)	1.56 (1.44–1.69)	1.56 (1.44–1.70)
Diameter		1.31 (1.24–1.38)	1.19 (1.11–1.29)	1.28 (1.17–1.39)
	Model 2			
IMT		1.46 (1.37–1.54)	1.45 (1.33–1.57)	1.46 (1.35–1.59)
Diameter		1.19 (1.13–1.27)	1.17 (1.08–1.26)	1.23 (1.12–1.34)
	Model 3			
IMT		1.42 (1.33–1.50)	1.41 (1.30–1.53)	1.43 (1.32–1.56)
Diameter		1.11 (1.04–1.18)	1.12 (1.03–1.22)	1.10 (1.01–1.21)

IMT = intima-media thickness.

*Odds ratios for 1 standard deviation increments of intima-medial thickness (IMT), diameter, wall area (calculated as described in the methods).

^†^Level of adjustment: Model 1 includes the vascular measure(s) only; Model 2 adds age, race, gender (for the full sample), and height; Model 3 includes age, gender (for the full sample), cigarette years, body mass index, blood pressure medication use, systolic and diastolic blood pressures, LDL- and HDL-cholesterol, white blood count, and cholesterol medication use.

For prospective studies, the median and maximum follow-up times were 5.9 and 11.7 years respectively. The strength of the unadjusted, prospective associations for baseline RCCA IMT (HR = 1.43), diameter (HR = 1.37), and wall area (HR = 1.50) (Table 3) were weaker than the comparable cross-sectional associations (Table 2), but each of the vascular parameters was statistically significantly associated with carotid atherosclerosis progression at all levels of adjustment in the single vascular models used to evaluate the full sample and the gender subsets (Table 3). Included together in the same prospective models, both IMT and diameter remained statistically associated with incident carotid lesions (progression) at all levels of adjustment of the full sample, and after basic adjustment (model 2) in the gender subsets. Diameter's gender-specific associations with carotid atherosclerosis progression after adjustment for IMT and risk factors were similar in magnitude to the cross-sectional associations for men and women, but in the smaller prospective sample, the diameter associations were of borderline statistical significance. To evaluate whether unidentified plaques in the RCCA at baseline contributed to the positive association between baseline diameter and carotid atherosclerosis progression, we did a sensitivity analysis where we evaluated the association between exam 1 vascular measures in longitudinal models restricted to persons without RCCA plaques at the second exam. RCCA diameter remained statistically significantly related to carotid atherosclerosis progression in the overall sample with only modest attenuation of the strength of association (Table 3 restricted models).

**Table 3:** Hazards ratios and 95% confidence intervals* for progression to carotid atherosclerotic lesions associated with each standard deviation increment of B-mode ultrasound right common carotid artery (RCCA) measures, Atherosclerosis Risk in Communities Limited Access Data (ARICLAD), 1987–1998.

		Hazards Ratios (95% Confidence Intervals)*		
RCCA Measure	Adjustment Level^†^	Full Sample	Men	Women
		N = 4845	N = 1850	N = 2995
IMT	Model 1	1.43 (1.33–1.53)	1.38 (1.24–1.53)	1.40 (1.27–1.54)
	Model 2	1.30 (1.20–1.40)	1.29 (1.16–1.44)	1.30 (1.18–1.44)
	Model 3	1.23 (1.14–1.32)	1.23 (1.10–1.37)	1.23 (1.12–1.36)
	Model 4	1.23 (1.14–1.32)	1.26 (1.13–1.41)	1.22 (1.11–1.35)
	Restricted^‡^	1.19 (1.11–1.28)	1.20 (1.08–1.36)	1.20 (1.09–1.32)
Diameter	Model 1	1.37 (1.28–1.46)	1.27 (1.14–1.40)	1.36 (1.23–1.50)
	Model 2	1.25 (1.17–1.35)	1.23 (1.11–1.36)	1.29 (1.16–1.42)
	Model 3	1.17 (1.08–1.25)	1.16 (1.05–1.29)	1.17 (1.06–1.30)
	Model 4	1.16 (1.08–1.25)	1.16 (1.04–1.30)	1.17 (1.05–1.29)
	Restricted^‡^	1.14 (1.05–1.23)	1.12 (1.00–1.25)	1.16 (1.05–1.29)
Wall area	Model 1	1.50 (1.40–1.61)	1.40 (1.27–1.56)	1.50 (1.36–1.66)
	Model 2	1.36 (1.26–1.47)	1.32 (1.19–1.47)	1.41 (1.26–1.57)
	Model 3	1.26 (1.17–1.36)	1.25 (1.12–1.39)	1.29 (1.16–1.43)
	Model 4	1.26 (1.17–1.36)	1.27 (1.14–1.42)	1.28 (1.15–1.42)
	Restricted^‡^	1.22 (1.13–1.32)	1.21 (1.08–1.36)	1.26 (1.13–1.40)
	Model 1			
IMT		1.29 (1.20–1.39)	1.31 (1.17–1.46)	1.30 (1.17–1.44)
Diameter		1.25 (1.17–1.34)	1.16 (1.04–1.29)	1.23 (1.11–1.36)
	Model 2			
IMT		1.23 (1.14–1.33)	1.23 (1.10–1.38)	1.23 (1.11–1.37)
Diameter		1.17 (1.09–1.27)	1.15 (1.03–1.28)	1.20 (1.08–1.34)
	Model 3			
IMT		1.19 (1.10–1.28)	1.19 (1.07–1.33)	1.20 (1.08–1.32)
Diameter		1.11 (1.03–1.19)	1.11 (0.99–1.23)	1.11 (0.99–1.23)
	Model 4			
IMT		1.19 (1.11–1.29)	1.23 (1.09–1.38)	1.19 (1.07–1.31)
Diameter		1.10 (1.02–1.19)	1.10 (0.98–1.23)	1.11 (0.99–1.23)
	Restricted^‡^			
IMT		1.16 (1.08–1.25)	1.18 (1.05–1.33)	1.17 (1.05–1.29)
Diameter		1.09 (1.01–1.18)	1.07 (0.95–1.20)	1.11 (1.00–1.24)

IMT = intima-media thickness.

*Hazards ratio for carotid atherosclerosis progression associated with each standard deviation (SD) increment of the vascular measure(s) (IMT, diameter, calculated circular wall area, or IMT and diameter) estimated using SAS LIFEREG procedure using interval censoring.

^†^Model 1 includes the vascular measure(s) only; Model 2 adds age, race, sex, and height; Model 3 includes age, (gender only in full data set), cigarette years of smoking, BMI, systolic and diastolic blood pressures, LDL-cholesterol, and white blood count. Model 4 includes all 20 covariates and was limited to the subset with complete data (total n = 4791; n = 1823 for men, and n = 2968 for women).

^‡ ^Restricted Model 4 estimates were made in a subset (total n = 4593; n = 1736 for men, and n = 2857 for women) that excluded persons who developed plaques/shadowing in the RCCA by the first follow-up examination.

Sequential adjustment indicates there is overlap in the excess risk explained by IMT and diameter (Table 3). For example, the hazard ratio for diameter was reduced from 1.37 to 1.25 after inclusion of IMT in the model. Adjusting for risk factors, but not IMT, reduced the hazard ratio from 1.37 to 1.17. Adjustment for both IMT and risk factors reduced the hazard ratio to 1.11. Similarly, IMT risk was reduced after adjusting for diameter and risk factors, but with a stronger risk remaining (HR = 1.19).

**Figure 2:**
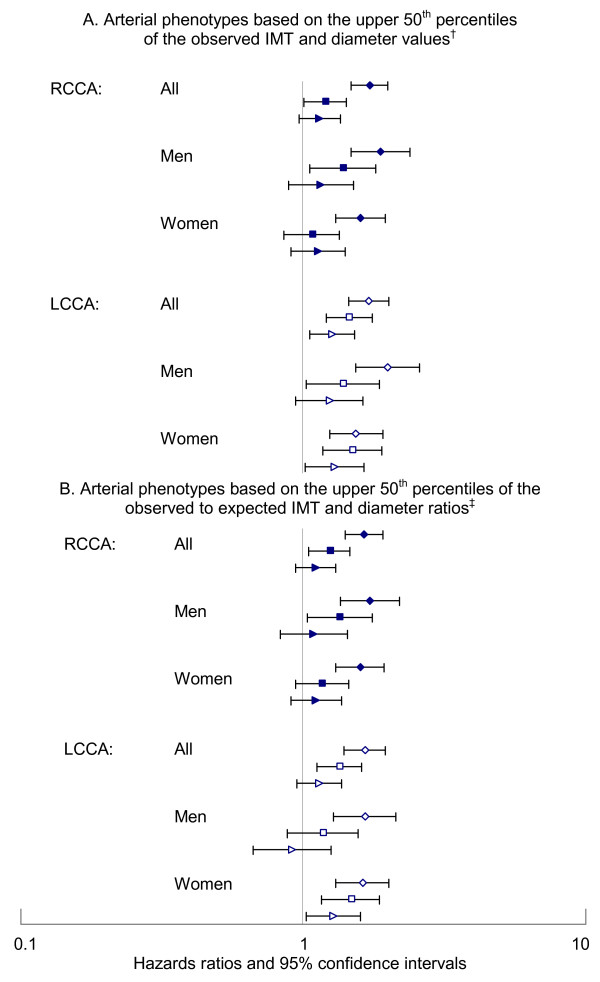
Adjusted* hazards ratios for progression to carotid atherosclerotic lesions for baseline common carotid artery phenotypes^†‡^. *Controlled for age, race and gender. ^†‡ ^Isolated or combined IMT and diameter enlargement based on A) observed measures and B) observed/expected ratios. Diamond = diameter and IMT enlarged. Square= isolated IMT enlargement. Triangle = isolated diameter enlargement. Solid = right and open = left.

When categorized by the median values of RCCA IMT and diameter, 32.0% of participants had both IMT and diameter enlarged, 18.4% had isolated IMT enlargement, 17.8% had isolated diameter enlargement, and 31.9% had neither enlarged while categorization based on the RCCA 50^th ^percentile observed/expected ratios produced proportions of 30.9%, 19.1%, 19.1%, and 30.9% respectively. These two methods of cross-classification varied significantly with 83.7% agreement as to having both IMT and diameter enlarged, 83% as to having neither enlarged, and 79–80% as to having only one parameter enlarged (p < 0.0001). However, in general, persons having enlargement of both RCCA diameter and IMT had the greatest risk of carotid atherosclerosis progression (risk of incident plaques) (Figure 2). Controlling for age, race, and sex, persons with both RCCA IMT and diameter in the upper 50^th ^percentiles of the sample had significantly increased risk of developing a readily identifiable carotid lesion compared to persons having neither abnormality and to persons having just one of the parameters enlarged. For women, isolated enlargement of RCCA IMT or diameter (measure in the upper 50^th ^percentile) did not produce a significant increase in risk of developing a carotid lesion; but for men, isolated RCCA IMT enlargement did result in significantly increased risk of developing a carotid lesion compared to men with neither parameter enlarged. Similarly, when enlargement was based on the upper 50^th ^percentiles of the ratios of the observed/expected RCCA carotid parameters produced qualitatively similar results.

Substituting LCCA parameter values in the prospective statistical models (N = 4187) produced results similar to those for the RCCA measures after adjusting for demographic factors and height (LCCA wall area HR = 1.37, 95% CI = 1.27–1.49; IMT HR = 1.30, 95% CI = 1.20–1.41; diameter HR = 1.26, 95% CI = 1.17–1.37) and after adjusting for 20 risk factors (LCCA wall area HR = 1.25, 95% CI = 1.14–1.35; LCCA IMT HR = 1.20, 95% CI = 1.11–1.30, LCCA diameter HR = 1.15, 95% = 1.06–1.25). Using LCCA values to define IMT and diameter enlargement, produced age-, race-, and gender-adjusted associations qualitatively similar to those for the RCCA (Figure 2) with the strongest risk of developing carotid lesions generally occurring among those with both LCCA IMT and external diameter enlargement.

Using the c-statistic to indicate model discrimination for the existence of carotid lesions, wall area led to a slight improvement in model discrimination (c-statistics for wall area: all 0.650, men: 0.636, women 0.633) compared to IMT (c-statistics for IMT: all 0.635, men 0.631, women 0.623). However, after adjusting for age, race, height and gender essentially no difference in risk discrimination remained (not shown).

## Discussion

Arterial wall thickening and diameter enlargement are intimately related with the anatomic changes generally proceeding in tandem which produces a complex relationship between the two parameters and atherosclerosis. The current study confirmed the overlapping atherosclerosis information provided by risk factors, wall thickness, and external diameter. More importantly, this study provides striking evidence that the arterial phenotype of co-existent wall thickening and diameter enlargement poses the greatest risk of atherosclerosis progression. The study proposes a method for determining "normal" artery parameters that may have general relevance to the classification of arterial structure.

Since wall thickening and arterial remodeling do not generally proceed independently but are linked by adaptive responses [33,35,44], IMT and diameter provide overlapping information in regards to the risk of atherosclerosis progression which is clearly evident from the sequential model adjustments. Only a modest independent relationship between the continuous diameter measure and atherosclerosis progression remained after adjustment for both wall thickness and traditional risk factors. This model improvement could be merely because diameter improves model calibration[45] or because the diameter reflects anatomic features that have an auto-catalyzing effect such as wall inflammation [23,36,46], or because diameter reflects a risk factor/genetic milieu with a generally greater propensity for progression, but separating these possibilities was not part of this study.

As reviewed, both resistance arteries [20] and larger conduit arteries [21,47] are subject to anatomic changes that can be categorized based on diameter and wall thickness in multiple ways as different arterial phenotypes [18,20-23]. Kiechl et al found that plaques developed preferentially at sites where the IMT was greater than the 50^th ^percentile [48] Our study extends Kiechl's study by showing that the RCCA phenotype with both diameter and IMT in the upper 50^th ^percentile had a significantly greater propensity for progression to definitive carotid lesions than when only IMT was enlarged. Isolated RCCA diameter enlargement generally had an even lower risk than isolated wall thickening, but the disparity was not statistically different. In a recent clinical study, a positive remodeling index was significantly related to an increase in diffuse in-stent restenosis [46]. Our results seem to suggest that diameter enlargement in the presence of wall thickening indicates some fundamental differences from isolated wall thickening. Just as atherosclerotic plaques with expansive remodeling are found to have an inflammatory component [36,49], wall thickening with expansive remodeling may also have a greater inflammatory component [50] or possibly a different genetic susceptibility than walls that do not exhibit expansive remodeling. Also, since the ARIC definition of CCA plaques required the wall to be at least 1.5 mm in thickness, smaller plaques would be missed. An alternate explanation for the lower risk of atherosclerosis progression among persons with only IMT thickening could be that the latter group included persons with non-atherosclerotic thickening such as response to hypertension with lower flow [18,20]. Evaluating reasons for the different arterial phenotypes is beyond the scope of the present study.

This study also presents a possible methodological improvement in defining arterial phenotypes. Defining what arterial diameter is normal has been problematic [21,22] with most recent definitions being based on adjacent reference arteries not displaying an atherosclerotic plaque [21,22]. However, it is widely recognized that sites free of local lesions can have a generalized dilation response [51]. A recent assessment of static and serial coronary artery remodeling clearly showed that cross-sectional comparison of sites with atherosclerotic lesions to a reference artery could result in misclassification of plaques as having constrictive rather than expansive remodeling [52]. Our study defined the normal IMT and diameter values for each person based on the common carotid arteries of men and women who were free of both major atherosclerotic disease and of major risk factors with the gender-specific "normal" values being estimated for each person's height, age, and race. Thus, the expected IMT and diameter values to which the observed values are compared are likely to be free of the effect of major risk factors and so represent ideal values expected for someone of similar age, gender, height, and race. This method can be used for other arterial sites where disease-free and risk factor-free values are available and could be used to identify diffuse remodeling, absence of remodeling, and constrictive remodeling.

Wall area provides a composite measure of IMT and diameter. However, while wall area did provide a modestly stronger association with carotid atherosclerosis progression than IMT, wall area will not distinguish the different arterial phenotypes that may be important in understanding atherosclerosis progression.

This study has certain limitations. In the study of incident carotid atherosclerotic lesions (atherosclerosis progression), the use of the ARIC plaque definition requiring a thickness of at least 1.5 mm, could have resulted in smaller plaques being missed at baseline. Others have shown that pre-existing plaques predicted development of new plaques and progression of existing plaques [48]. So, we cannot be sure that diameter enlargement at baseline was not because of non-diagnosed plaques that had produced expansive remodeling. Even the sensitivity analysis that excluded persons with RCCA lesions at exam 2, cannot exclude this possibility.

The rate of focal arterial remodeling in atherosclerosis depends upon initial lumen size [53] which complicates the use of diameter as an indicator of atherosclerosis. Thus, body stature and age which are correlated with arterial diameter [12,54], could impact the association between diameter and atherosclerosis. Also, there appears to be a limit to arteries' ability to enlarge in response to wall thickening [34] which could limit diameter's usefulness as an indicator of atherosclerosis among the elderly. Our use of a classification of enlargement based on the observed to expected arterial parameter diminished some of these concerns. Our results support the contention [9] that considering arterial diameter as well as wall thickness is essential in understanding the atherosclerotic process.

Our results may not be representative of the ARIC cohort as participants without complete data on carotid atherosclerosis were excluded. Also, the reader- and trend-adjusted IMT values used in many ARIC manuscripts were not available for these analyses. A change of ultrasound equipment occurred during the third exam and the ultrasound protocol was simplified from three views at baseline to a single view at exams 3 and 4. This could have contributed to differences in plaques/shadowing recognition between early and later exams. While diameter measurements may vary depending upon the scan view, variability was minimized by using measurements from the view with defined structures. Since vascular measurements did not intentionally exclude plaques, IMT and diameter measurements at baseline could reflect both adaptive response and atherosclerosis as discussed above.

In conclusion, B-mode ultrasound-measured RCCA diameter is associated with the progression of atherosclerosis in the carotid arterial system. A method devised to define the reference CCA parameters may remove some previous methodological limitations. Our study suggests that presence of both external RCCA diameter enlargement as well as wall thickening may indicate a high risk arterial phenotype. Future studies should investigate factors relating to the different arterial phenotypes as well as how arterial diameters relate to local or carotid system changes.

## Competing interests

The author(s) declare that they have no competing interests.

## Authors' contributions

MLE conceived and designed the study, contributed the method for comparing observed to expected vascular measures, drafted and revised the manuscript.

ZB and DJC provided input into the analysis plan and ZB performed and interpreted many analyses and they and KMR contributed to multiple drafts of the manuscript. RET and JLM provided input into overall concepts of the study, and contributed to multiple drafts of the manuscript.

All authors have agreed to submission of the final manuscript.
